# Supplementary material for: Acute effect of transcutaneous auricular vagus nerve stimulation on cardiac vagal activity in men living with HIV: A proof-of-concept clinical trial
Source: PLoS One. 2025 Aug 19;20(8):e0326793. doi: 10.1371/journal.pone.0326793 (PMC12364314; doi:10.1371/journal.pone.0326793)
Supplement: Attachment — Submitted filename: Response to Reviewers.docx [file pone.0326793.s003.docx]

**Rebuttal letter**

Dear Editor,

Dr. Emily Chenette

PLOS ONE

We are pleased to present here our revised version of the manuscript now entitled **“****Acute effect of transcutaneous auricular vagus nerve stimulation on cardiac vagal activity in men living with HIV: a proof-of-concept clini-cal trial.”** (PONE-D-24-18721.R1) which was submitted to PONE.

The authors would like to thank the Journal and its Editorial Board for allowing us to proceed with the review process. We would like to thank the reviewers for their positive feedback and detailed comments and suggestions on the manuscript. They have identified important aspects of our paper that required relevant amendments, and their evaluations have greatly improved the quality of the manuscript.

We have carefully reviewed all the reviewers’ suggestions and provided a detailed, point-by-point response to each comment. To facilitate the review process, we have highlighted the reviewers’ comments in **bold**, our responses in blue, and any additions or modifications to the main manuscript in yellow.

Response structure:

- **Reviewer’s comment**
- Our response
- Added or modified text in the manuscript

In the main manuscript file, all changes have also been highlighted in yellow for easy identification.

Yours sincerely,

The authors.

**REVIEWER 1**

**Reviewer #1: Review of PLOS ONE PONE-D-24-18721 manuscript July 2024**

‘Acute effect of transcutaneous auricular vagus nerve stimulation on cardiac vagal
activity in men living with HIV: a randomized clinical trial’.

The main aim of this research article is clear. The aim is to test taVNS in HIV persons to see if it affects vagal activity. Apart from improving vagal activity, could taVNS lower sympathetic activity in HIV persons or healthy persons?

The aim is novel, as this data has not been previously collected. The Introduction and Methods section have appropriate detail. Some more details could be added to the Methods.

Results are relatively clear and the data is reasonably well presented with tables and graphs. Although some sections could be better written, especially on GMM data.
The Discussion has a satisfactory level of critical analysis, although more can be added on the time changes, could they be related to a repeated effect. The significance of the study was given, and comparisons made with previous studies, and an emphasis on the applied nature of this research.

There are no major issues with the methodology or data analysis. This manuscript could be better presented. The language and grammar is acceptable, some improvements in grammar could be made. References are correct but a more concise number could be used. Ref 44 and 45 are the same.

**Response:** Dear reviewer, the authors would like to thank you for all the invaluable time and energy you devoted to revising the present manuscript as well as for seeing its potential contribution. We have addressed all your comments and suggestions point-by-point below and have amended the text to accommodate your suggestions. All changes are marked in yellow in the main manuscript. We hope we have met your expectations and that the paper is now deemed suitable for publication. The duplicate references have been removed throughout the text.

**Corrections/suggestions for the authors are mentioned below:**

**Abstract**

1. **Line 33-34. Clearly state if there was no significant difference in parameters between active and placebo.**

**Response:** Thank you very much for the suggestion. We have clarified the absence of differences. The sentence now reads as follows:

“No significant changes in vmHRV parameters were observed over time or between conditions. These findings suggest that an acute taVNS session does not modulate cardiac vagal activity in people living with HIV.” (Abstract, L 33-35).

**Intro**

1. **Line 58. Should ‘autonomic peripheral system’ be the ‘autonomic nervous system’?**

**Response:** Thank you for spotting this erroneous nomenclature. We change the text to the correct nomenclature.

2. **Line 60. Add ‘could counteract some of these impairments’**

**Response:** Thank you very much! Another reviewer also suggested a change in this paragraph. They asked to make it clear which therapy we were referring to. After making the revisions, the text now reads as follows:

“These effects may exacerbate the impairments caused by HIV's direct impact on the central nervous, autonomic, and cardiovascular systems, thereby increasing the risk of developing cardiovascular disease [1,6–9]. Transcutaneous Auricular Vagus Nerve Stimulation (taVNS) is a non-pharmacological adjunct therapy with the potential to help mitigate some of the long-term adverse effects of antiretroviral therapy, particularly regarding cardiac autonomic function) [10].” (Intro, L 56-62).

3. **Line 83. What type of vagal activity was used?**

**Response:** Dear reviewer, we used cardiac vagal activity. After your observation, we amended the text as follows:

“In accordance, lower resting HRV indices in people with HIV has been associated with lower cardiac vagal activity compared to people without the HIV [19,20].” (Intro, L 82-84).

4. **Line 89. Which part of the auricle? The whole external ear? This detail has been given in the methods, but could be added here for greater clarity.**

**Response:** We used the cymba conchae. We have added this information to the text as follows:

“The mechanism of action of taVNS on cardiac vagal activity can be explained by the presence of vagal nerve endings in the human auricle, especially in the regions of the cymba conchae and tragus.” (Intro, L 88-90).

**Methods**

1. **Line 110. MLHIV meaning? Were females not available?**

**Response 1:** Thank you for your comment. The meaning of MLHIV is: Men living with HIV. We included the meaning of the acronym in the text as follows:

“Given that taVNS may positively influence vagal tone, this study aimed to investigate the acute effect of taVNS on cardiac vagal activity in men living with HIV (MLHIV).” (Intro, L 105-107).

As for the inclusion of males’ participants only, we have also added the reasons to the manuscript as follows:

“The study was restricted to male participants for three reasons: First, sex differences in heart rate variability are evident and this could make the results confusing [33]. Second, there is a difference in the autonomic response between the sexes during electrical stimulation of the vagus nerve [34]. Third, women can show fluctuations in HRV, with notable changes between the follicular and luteal phases, mainly due to variations in progesterone levels [35,36].” (Methods, L 114-119).

**REFERENCES**

- Koenig, J., & Thayer, J. (2016). Sex differences in healthy human heart rate variability: A meta-analysis. *Neuroscience & Biobehavioral Reviews*, 64, 288-310. <https://doi.org/10.1016/j.neubiorev.2016.03.007>.
- Veiz, E., Kieslich, S., Staab, J., Czesnik, D., Herrmann-Lingen, C., & Meyer, T. (2021). Men Show Reduced Cardiac Baroreceptor Sensitivity during Modestly Painful Electrical Stimulation of the Forearm: Exploratory Results from a Sham-Controlled Crossover Vagus Nerve Stimulation Study. *International Journal of Environmental Research and Public Health*, 18. https://doi.org/10.3390/ijerph182111193.
- Schmalenberger, K., Eisenlohr-Moul, T., Jarczok, M., Eckstein, M., Schneider, E., Brenner, I., Duffy, K., Schweizer, S., Kiesner, J., Thayer, J., & Ditzen, B. (2020). Menstrual Cycle Changes in Vagally-Mediated Heart Rate Variability Are Associated with Progesterone: Evidence from Two Within-Person Studies. *Journal of Clinical Medicine*, 9. <https://doi.org/10.3390/jcm9030617>.
- Schmalenberger, K., Eisenlohr-Moul, T., Würth, L., Schneider, E., Thayer, J., Ditzen, B., & Jarczok, M. (2019). A Systematic Review and Meta-Analysis of Within-Person Changes in Cardiac Vagal Activity across the Menstrual Cycle: Implications for Female Health and Future Studies. *Journal of Clinical Medicine*, 8. <https://doi.org/10.3390/jcm8111946>.

2. **Line 153. Why was the left ear used?**

**Response:** Thank you for your comment. We decided to stimulate the left ear for some reasons that were also added to the text after your comment. Please see the new sentence of the manuscript below:

“The choice of the left ear for auricular stimulation of the vagus nerve was supported by anatomical and functional evidence indicating a greater density of innervation, significant neuroprotective effects and greater consistency in the therapeutic response [21,41,42]. In addition, for greater comparability we followed the consensus document by Farmer et al (2021) [11] which highlights the predominance of stimulation protocols in the left ear.” (Methods, L 168-173).

**REFERENCES**

- Butt, M., Albusoda, A., Farmer, A., & Aziz, Q. (2019). The anatomical basis for transcutaneous auricular vagus nerve stimulation. *Journal of Anatomy*, 236. <https://doi.org/10.1111/joa.13122>.
- Yakunina, N., Kim, S., & Nam, E. (2017). Optimization of Transcutaneous Vagus Nerve Stimulation Using Functional MRI. *Neuromodulation: Technology at the Neural Interface*, 20. https://doi.org/10.1111/ner.12541.
- Keute, M., Machetanz, K., Berelidze, L., Guggenberger, R., & Gharabaghi, A. (2021). Neuro-cardiac coupling predicts transcutaneous auricular vagus nerve stimulation effects. *Brain Stimulation*, 14, 209-216. <https://doi.org/10.1016/j.brs.2021.01.001>.
- Farmer, A., Strzelczyk, A., Finisguerra, A., Gourine, A., Gharabaghi, A., Hasan, A et al. (2021). International Consensus Based Review and Recommendations for Minimum Reporting Standards in Research on Transcutaneous Vagus Nerve Stimulation (Version 2020). *Frontiers in Human Neuroscience*, 14. <https://doi.org/10.3389/fnhum.2020.568051>.

3. **Line 161. How many subjects had a lowered intensity?**

**Response:** Of the 21 participants, 4 had a reduction in intensity, 1 participant had a reduction up to the perception threshold. Another 3 participants had intensities above the perception threshold by 118%, 125% and 162%. In the sham condition, 2 participants requested a reduction. The sentence now reads as follows:

“In both conditions (taVNS and sham), the researcher remained beside the stimulator throughout the session to intervene in case any participant reported discomfort. During the taVNS condition, intensity adjustments were made for four participants who reported discomfort: one participant had the intensity reduced to the perceptual threshold, while the other three had the intensity adjusted to levels still above the perceptual threshold (118%, 125%, and 162%, respectively). In the sham condition, although the stimulator was turned off, two participants requested a reduction in intensity because they believed they were feeling stimulation. (Methods, L 200-207).

4. **Line 164. So was the intensity at 0 mA for placebo?**

**Response:** Exactly. We decided to adopt this sham protocol for the reasons explained below, which was included into the main manuscript:

**“**The choice of the sham protocol is in line with previous studies that used the same ear region as the active condition to position the electrodes while the electrostimulator remained switched off [42,44,45]. There are protocols that use the ear lobule as an active sham [11] However, it is possible that stimulation in this region may not be entirely specific and could influence multiple neural pathways, leading to activation patterns similar to taVNS stimulation [21].” (Methods, L 194-199).

**REFERENCES**

- Keute, M., Machetanz, K., Berelidze, L., Guggenberger, R., & Gharabaghi, A. (2021). Neuro-cardiac coupling predicts transcutaneous auricular vagus nerve stimulation effects. *Brain Stimulation*, 14, 209-216. <https://doi.org/10.1016/j.brs.2021.01.001>.
- Couck, M., Cserjési, R., Caers, R., Zijlstra, W., Widjaja, D., Wolf, N., Luminet, O., Ellrich, J., Ellrich, J., Gidron, Y., & Gidron, Y. (2017). Effects of short and prolonged transcutaneous vagus nerve stimulation on heart rate variability in healthy subjects. *Autonomic Neuroscience*, 203, 88-96. <https://doi.org/10.1016/j.autneu.2016.11.003>.
- Clancy, J., Mary, D., Witte, K., Greenwood, J., Deuchars, S., & Deuchars, J. (2014). Non-invasive Vagus Nerve Stimulation in Healthy Humans Reduces Sympathetic Nerve Activity. *Brain Stimulation*, 7, 871-877. <https://doi.org/10.1016/j.brs.2014.07.031>.
- Farmer, A., Strzelczyk, A., Finisguerra, A., Gourine, A., Gharabaghi, A., Hasan, A et al. (2021). International Consensus Based Review and Recommendations for Minimum Reporting Standards in Research on Transcutaneous Vagus Nerve Stimulation (Version 2020). *Frontiers in Human Neuroscience*, 14. <https://doi.org/10.3389/fnhum.2020.568051>.
- Butt, M., Albusoda, A., Farmer, A., & Aziz, Q. (2019). The anatomical basis for transcutaneous auricular vagus nerve stimulation. *Journal of Anatomy*, 236. <https://doi.org/10.1111/joa.13122>.

5. **Line 188. Why was the last 10 minutes selected for HRV analysis, and not the first 10 minutes? This seems to contract Lines 196-197?**

**Response:** Thank you for the observation. We revised the text to improve its clarity. Additionally, we decided to modify the figure illustrating the HRV analysis moments to clearly indicate the exact time points where RR intervals were recorded and the segments selected for heart rate variability analysis. The updated text and picture can be seen below:

**“**A total of six 10-minute segments were extracted from the timeline for analysis: Moment 1 (baseline), Moments 2 and 3 during stimulation (0-10 min and 20-30 min), and Moments 4, 5, and 6 during recovery (30-40 min, 50-60 min, and 80-90 min). Within each 10-minute window corresponding to these moments, HRV was analyzed in the first 5 minutes across both conditions and for all participants to ensure better data comparability.” (Methods, L 249-253).

Fig 3 Timeline of recording and analysis of RR intervals. HRVA, heart rate variability analysis; PT, perception threshold; ER, earphone removal; VAS, visual analog scale.

6. **Line 198. State LH, HF and ratio assessed.**

**Response:** Dear reviewer, after considering the feedback from another reviewer, we decided to remove the analyses of other time-domain and frequency-domain measures from the tables, as they are not part of the primary outcome. We clarify that our intent was solely to investigate the variables rMSSD and pNN50, as they better represent cardiac vagal activity. The statement is supported by the references below.

**REFERENCES**

- Laborde, S., Mosley, E., & Thayer, J. (2017). Heart Rate Variability and Cardiac Vagal Tone in Psychophysiological Research – Recommendations for Experiment Planning, Data Analysis, and Data Reporting. *Frontiers in Psychology*, 8. <https://doi.org/10.3389/fpsyg.2017.00213>.
- Malik, M., Camm, A.J., Bigger, J.T., Breithart, G., Cerutti, S., Cohen, R., 1996. Heart rate variability: standards of measurement, physiological interpretation and clinical use. Task force of the European Society of Cardiology and the north American Society of Pacing and Electrophysiology. Circulation 93 (5), 1043–1065.

7. **Line 201. When was BP measured?**

**Response:** Thank you for the observation. Blood pressure was measured at baseline. We have clarified this in the text. The revised sentence can be read below:

**“**Systolic and diastolic blood pressures (SBP and DBP) were measured after 10 minutes of rest at baseline using an Ambulatory Blood Pressure Monitor CONTEC® ABPM50 (Contec Medical Systems - Hebei, China).” (Methods, L 257-259).

8. **Line 218. Use consistent terminology experimental/control or active/placebo.**

**Response:** Thank you very much. Following your observation, we have adopted the nomenclature taVNS (active) and sham throughout the text.

9. **Line 221. Could watching a video affect their HRV?**

**Response:** Thank you for your observation. We used “point of view” videos to distract participants during the stimulation and sham periods. Please note that being seated looking at a wall for long periods might be boring/tedious and could also direct their attention towards experiment details. We took care to present videos of the same style that would not evoke emotions capable of influencing HRV. Therefore, we did not expect any changes. However, after considering your observation, we recognized this as a limitation (please, see below), as we did not use empirically tested/validated videos as neutral.

“Participants in the study spent the entire session seated on a couch, watching POV-style videos of streets in different countries. Care was taken to select videos of the same style and content to avoid evoking emotions such as anger, sadness, or euphoria. However, this aspect was not controlled in the study, as the videos had not been empirically tested or validated as neutral.” (Limitations, L 511-515).

10. **Line 225. Line 188 says it was the last 10 minutes?**

**Response:** Thank you very much. With the changes made to the sentences in lines (Methods, L 249-253), we believe the text has become clearer. Consequently, the text on lines 286-292 was revised and can be seen in the following sentence:

“Following the selection of the video, RR interval recording commenced for a 10-minute baseline period. The recording was paused to determine the perception threshold. Subsequently, RR intervals were continuously recorded throughout the entire stimulation period (30 min) and recovery period (60 min). At the end of the stimulation period, the earbud-shaped electrode was removed from the participant’s ear, and the investigator presented the visual analog scale to the participant. The timeline of the stimulation session can be better visualized (Fig. 3).” (Methods, L 281-287).

11. **Line 229 and 231. Two Fig 3 captions are given and for the first one remove ‘This is the Fig 3’.**

**Response:** Thank you very much. The errors have been corrected.

12. **Line 257. Virtual or visual for pain scale?**

**Response:** Thank you very much. Visual scale. Error corrected.

**Results**

1. **Line 264. Height and weight are given in Table 1, but it is not mentioned how this was measured in the Materials section. Same for viral load and cART data.**

**Response:** Thank you very much for this observation. We have added the missing information to the text regarding these measurements:

“The participants attended three laboratory visits, each separated by 48 hours. During the initial visit, they participated in a structured anamnesis interview, which included questions about age, current antiretroviral therapy, diagnosis time, and the submission of a physician-authorized clinical report containing data from the most recent CD4 and viral load tests. Subsequently, body composition was assessed. Weight and height measurements were obtained using a digital scale with an integrated stadiometer (Welmy®), calibrated in accordance with the manufacturer's guidelines. Measurement protocols adhered to the standards established by standards for anthropometry assessment [52]. Body fat percentage and fat-free mass were assessed using dual-energy X-ray absorptiometry (DEXA) [53] with a GE Lunar Prodigy Advance 2015 device (GE Medical Systems, Madison, WI), and analyzed via Encore Version 15 software.” (Methods, L 262-272).

**RFERENCES**

- Norton, K. I. (2018). Standards for anthropometry assessment. In *Kinanthropometry and exercise physiology* (pp. 68-137). Routledge <https://doi.org/10.4324/9781315385662-4>.
- Petak, S., Barbu, C., Yu, E., Fielding, R., Mulligan, K., Sabowitz, B., Wu, C., & Shepherd, J. (2013). The Official Positions of the International Society for Clinical Densitometry: body composition analysis reporting. *Journal of clinical densitometry: the official journal of the International Society for Clinical Densitometry*, 16 4, 508-19. <https://doi.org/10.1016/j.jocd.2013.08.018>.

2. **Line 279 Use SDRR in place of SDNN. Are these values not very low? Normally they are within range of 20-80 ms.**

**Response:** Dear reviewer, after considering the feedback from another reviewer, we decided to remove the analyses of other time-domain and frequency-domain measures from the tables, as they are not part of the primary outcome. We clarify that our intent was solely to investigate the variables rMSSD and pNN50, as they better represent cardiac vagal activity. The statement is supported by the references below.

**REFERENCES**

- Laborde, S., Mosley, E., & Thayer, J. (2017). Heart Rate Variability and Cardiac Vagal Tone in Psychophysiological Research – Recommendations for Experiment Planning, Data Analysis, and Data Reporting. *Frontiers in Psychology*, 8. <https://doi.org/10.3389/fpsyg.2017.00213>.
- Malik, M., Camm, A.J., Bigger, J.T., Breithart, G., Cerutti, S., Cohen, R., 1996. Heart rate variability: standards of measurement, physiological interpretation and clinical use. Task force of the European Society of Cardiology and the north American Society of Pacing and Electrophysiology. Circulation 93 (5), 1043–1065.

3. **Line 297-307. These changes could be written more concisely.**

**Response:** Thank you for this observation. After reanalyzing the data, the results have been described as shown in the sentence below:

“The GEE revealed no significant effects of condition (rMSSD: χ²(1) = .049, *p* = .484; pNN50: χ²(1) = 1.160, *p* = .281), time (rMSSD: χ²(5) = 5.81, p = 0.325; pNN50: χ²(5) = 6.250, *p* = .283), or condition-by-time interaction (rMSSD: χ²(5) = .260, *p* = .998; pNN50: χ²(5) = .130, *p* = 1.000) for rMSSD or pNN50, indicating taVNS did not significantly alter these measures compared to sham over time. Effect sizes (Cohen’s *d*) for within-condition changes relative to baseline ranged from moderate to large for rMSSD and pNN50 in taVNS, and from small to moderate (rMSSD) or moderate to large (pNN50) in sham, with similar trends over time. Between-condition effect sizes were consistently small at each time point, with overall effect sizes of *d* = 0.52 (rMSSD) and *d* = 0.78 (pNN50), indicating moderate effects. Marginal effects of taVNS versus sham were nonsignificant for rMSSD (2.08 ms, 95% CI: -3.77 to 7.99 ms, Z = 0.69, *p* = .485) and pNN50 (2.23%, 95% CI: -1.84 to 6.37%, Z = 1.08, *p* = .282). Descriptive results are detailed in Table 3.” (Results, L 355-367).

4. **Line 310 typo.**

**Response:** Thank you. The error has been corrected.

**Discussion**

1. **Line 324. Use improve in place of increase, as SDRR would decrease if better and likewise ratio would go down, but HF would increase.**

**Response:** Dear reviewer, after considering the feedback from another reviewer, we decided to remove the analyses of other time-domain and frequency-domain measures from the tables, as they are not part of the primary outcome. We clarify that our intent was solely to investigate the variables rMSSD and pNN50, as they better represent cardiac vagal activity. The statement is supported by the references below.

**REFERENCES**

- Laborde, S., Mosley, E., & Thayer, J. (2017). Heart Rate Variability and Cardiac Vagal Tone in Psychophysiological Research – Recommendations for Experiment Planning, Data Analysis, and Data Reporting. *Frontiers in Psychology*, 8. <https://doi.org/10.3389/fpsyg.2017.00213>.
- Malik, M., Camm, A.J., Bigger, J.T., Breithart, G., Cerutti, S., Cohen, R., 1996. Heart rate variability: standards of measurement, physiological interpretation and clinical use. Task force of the European Society of Cardiology and the north American Society of Pacing and Electrophysiology. Circulation 93 (5), 1043–1065.

2. **Line 425. Would this limitation be countered by the fact other PWHIV studies have measured HRV.**

**Response:** Thank you for this observation. While we agree with the reviewer, we have chosen to maintain these limitations as we are not aware of other studies utilizing taVNS in people living with HIV.

3. **Line 396, 446, and 454. Typos.**

**Response:** Thank you. These errors have also been corrected.

**REVIEWER 2**

**Reviewer #2: Thank you for inviting me to review this paper it is an honour and a privilege to do so.**

On the topic of the paper itself, the paper focuses on autonomic neuromodulation through transcutaneous auricular vagus nerve stimulation (taVNS) in individuals living with HIV. The field of electrical modulation of the parasympathetic system (PNS) has experienced significant expansion in recent years due to the non-invasive nature of the procedure, which has little to no side effects. The authors are the first to apply this technique to the population of patients with HIV. They employed a fairly standard approach by conducting a crossover study that included both a sham control and single blinding. Their selected marker for assessing PNS activation was heart rate variability (HRV). This is a reasonable choice, as it is one of the very few indicators that have been shown to change in response to taVNS (when compared to sham), although only in the frequency domain using the LF/HF ratio (Hua et al., 2023).

The study which the paper describes is in itself fine. The quality and execution of the study is in line with other studies in this field. However, there are some key issues that need to be addressed. The introduction and discussion sections are inadequately presented. There should be improved coherence in the discussion of topics. Currently, topics are referenced multiple times in various contexts, at times in ways that are very confusing for the reader.

There are issues regarding blinding. Although the study is presented as single-blinded, the blinding issues are substantial enough that the study, in my view, cannot be classified as such (refer to major issues, line 165 in the manuscript). Furthermore, it is unclear which periods were used for HRV calculations as it is very poorly presented - if different periods were used to calculate HRV for each measurement, which was my understating from reading the methods, than the results are not comparable between participants and interventions (sham and taVNS). If this is indeed the case, the HRV calculations and the statistical analysis should be redone using the exact same five-minute periods for all participants. It may be somewhat acceptable (though not ideal) to choose arbitrary five-minute periods during baseline HRV assessment, but not during the stimulation phases and recovery, where changes in the dynamic of HRV are not only likely, but expected!

To summarize: The study was conducted accurately, but the presentation is flawed, as the patients were aware that one stimulation differed from the other, thus compromising the blinding process. Furthermore the calculation of HRV should be done on the exact same 5 min periods across all measurements. This issue must be rectified prior to publication. There exists a discrepancy in the number of HRV indices presented in the text and in the tables, this has impact on the statistical evaluation, since the correction for multiple testing will vary according to the number of hrv indices used. The presentation requires improvement, as there are moments when it is unclear what the authors intended to convey to the audience.

**Major issues:**

**165 - Consideration of the reviewer; regarding sham stimulation: Sham stimulation is a tricky one in the realm of taVNS and I do understand the struggle, there is no ideal solution. Despite that, two limitations of the sham design in this study need to be addressed. 1) The first consideration stems from the fact that during the sham intervention the participants received a tiny amount of stimulation from when their personal stimulation current was being determined, which may or may not have an impact on the PNS. 2) The second consideration is related to the fact that participants were told that the intensity would be lowered and the experience of stimulation would be decreased during the sham intervention but they weren’t told the same during the taVNS intervention. This way the participants received information on what to expect before the stimulation. Additionally if they were briefed beforehand on receiving sham and taVNS on different days they could very easily deduce which is which.**

**Response:** Dear Reviewer, we sincerely thank you for your consideration and for allowing us the opportunity to elaborate further on the rationale behind the study's protocol choices.

To determine the stimulation intensity for the active condition, it is essential to identify the perception threshold for each participant before each session (Badran et al. 2019). For the sham condition, we adopted an inactive simulation model using the same auricular region as the taVNS active condition. This approach is widely employed in the literature (Gerges et al. 2024). We were particularly cautious about the potential issues that might arise from using active sham stimulation in the earlobe, such as participants questioning the change in the stimulated region. It´s also possible that this region may not be entirely specific and could influence multiple neural pathways, leading to activation patterns similar to taVNS stimulation (Butt et al. 2020). Our protocol closely aligns with those utilized in previous studies (Clancy et al. 2014; De Couck et al. 2017; Keute et al. 2021).

We acknowledge the theoretical possibility that the brief stimulation used to determine the perception threshold could have residual effects on the nervous system. However, the existing literature does not provide conclusive evidence that such brief stimulation would generate measurable physiological effects, particularly when compared to the 30-minute duration of active stimulation in our protocol.

Moreover, our statistical analyses did not reveal any significant interaction effects, differences between conditions, or changes over time for any of the variables analyzed. This lack of significant differences suggests that any potential residual effect from the perception threshold determination did not significantly influence the outcomes evaluated.

We have made adjustments to the manuscript to clarify this point further. The changes can be found below:

“To determine the perceptual threshold, a trained researcher attached the electrodes to the participant's left ear and then gradually adjusted the intensity of the stimulator, starting at 0 mA. The intensity was increased or decreased until the participant reported a slight sensation of vibration. The perceptual threshold was confirmed after the participant gave four consecutive positive responses to the same intensity. This procedure was standardized and applied in both conditions, taVNS and sham. After identifying the perceptual threshold, the stimulator was turned off to calculate the percentage of intensity to be applied during the session. At this point, the participant was informed that the stimulation session would last 30 minutes. In the taVNS condition, the stimulator was turned on, and an intensity corresponding to 200% of the perceptual threshold was applied. In the sham condition, the electrodes remained in place, but the stimulator was turned off. The choice of the sham protocol is in line with previous studies that used the same ear region as the active condition to position the electrodes while the electrostimulator remained switched off [42,44,45]. There are protocols that use the ear lobule as an active sham [11] However, it is possible that stimulation in this region may not be entirely specific and could influence multiple neural pathways, leading to activation patterns similar to taVNS stimulation [21]”. (Methods, L 183-199).

Regarding the second consideration, it is important to note that participants were not told there would be a sham condition nor what to expect in both sessions. We apologize for any confusion caused by the text of the first version of the manuscript. In the current version we clearly informed that participants were unaware of the existence of two different conditions (taVNS and sham) as below:

**“**In both conditions (taVNS and sham), the researcher remained beside the stimulator throughout the session to intervene in case any participant reported discomfort. During the taVNS condition, intensity adjustments were made for four participants who reported discomfort: one participant had the intensity reduced to the perceptual threshold, while the other three had the intensity adjusted to levels still above the perceptual threshold (118%, 125%, and 162%, respectively). In the sham condition, although the stimulator was turned off, two participants requested a reduction in intensity because they believed they were feeling stimulation.

To minimize potential bias in blinding, all participants were informed in both conditions that the stimulator would remain on during the session, even if they expressed doubts about perceiving the stimulus”. (Methods, L 200-207).

**REFERENCES**

- Badran, B., Yu, A., Adair, D., Mappin, G., Devries, W., Jenkins, D., George, M., & Bikson, M. (2019). Laboratory Administration of Transcutaneous Auricular Vagus Nerve Stimulation (taVNS): Technique, Targeting, and Considerations. *Journal of visualized experiments: JoVE*, 143. <https://doi.org/10.3791/58984>.
- Gerges, A., Williams, E., Hillier, S., Uy, J., Hamilton, T., Chamberlain, S., & Hordacre, B. (2024). Clinical application of transcutaneous auricular vagus nerve stimulation: a scoping review. *Disability and rehabilitation*, 1-31. <https://doi.org/10.1080/09638288.2024.2313123>.

Keute, M., Machetanz, K., Berelidze, L., Guggenberger, R., & Gharabaghi, A. (2021). Neuro-cardiac coupling predicts transcutaneous auricular vagus nerve stimulation effects. *Brain Stimulation*, 14, 209-216. <https://doi.org/10.1016/j.brs.2021.01.001>.

- Couck, M., Cserjési, R., Caers, R., Zijlstra, W., Widjaja, D., Wolf, N., Luminet, O., Ellrich, J., Ellrich, J., Gidron, Y., & Gidron, Y. (2017). Effects of short and prolonged transcutaneous vagus nerve stimulation on heart rate variability in healthy subjects. *Autonomic Neuroscience*, 203, 88-96. <https://doi.org/10.1016/j.autneu.2016.11.003>.
- Clancy, J., Mary, D., Witte, K., Greenwood, J., Deuchars, S., & Deuchars, J. (2014). Non-invasive Vagus Nerve Stimulation in Healthy Humans Reduces Sympathetic Nerve Activity. *Brain Stimulation*, 7, 871-877. <https://doi.org/10.1016/j.brs.2014.07.031>.

**195 – Author: “The 5-minute periods with the most stable heart rate were used for analysis” Consideration of the reviewer: I am not entirely sure about the length of periods used for HRV calculations. Typically, HRV calculations are performed over 5-minute periods. However, in line 195, you refer to 5-minute periods, whereas in line 197, you indicate 10-minute periods. My best guess is that within a 10 minute window an arbitrary 5 minute period was used for HRV calculation that best met some standard. If that is the case, it is quite problematic since HRV calculations are then not comparable between participants and interventions.**

**Response:** We fully understand your observation and have decided to reanalyze the HRV recordings. To ensure standardization across all participants, we selected the same 5-minute periods within each of the six 10-minute windows. Specifically, we adopted the first 5 minutes of each 10-minute window for HRV calculations. Based on your comment, we also realized that our figure could be more informative, so we have made adjustments accordingly. The updated text and picture can be seen below:

**“**A total of six 10-minute segments were extracted from the timeline for analysis: Moment 1 (baseline), Moments 2 and 3 during stimulation (0-10 min and 20-30 min), and Moments 4, 5, and 6 during recovery (30-40 min, 50-60 min, and 80-90 min). Within each 10-minute window corresponding to these moments, HRV was analyzed in the first 5 minutes across both conditions and for all participants to ensure better data comparability.” (Methods, L 249-253).

Fig 3 Timeline of recording and analysis of RR intervals. HRVA, heart rate variability analysis; PT, perception threshold; ER, earphone removal; VAS, visual analog scale.

**Table 2 – Consideration of the reviewer: Up until now there was no mention of other than RMSSD and pNN50 HRV indices, but in this table others are listed as well (SDNN, HF, LF, LF/HF)! If indeed these indices were calculated (based on the fact that statistics for them were presented in the aforementioned table) a correction for multiple testing needs to be done in the statistical analysis.**

**Response:** Thank you very much for yet another excellent observation. We have addressed your considerations and reanalyzed all statistical data, focusing exclusively on rMSSD and pNN50. We chose to present only these two indices of variability as they better reflect cardiac vagal activity, aligning with the primary objective of the study. The statement is supported by the references below.

**REFERENCES**

- Laborde, S., Mosley, E., & Thayer, J. (2017). Heart Rate Variability and Cardiac Vagal Tone in Psychophysiological Research – Recommendations for Experiment Planning, Data Analysis, and Data Reporting. *Frontiers in Psychology*, 8. <https://doi.org/10.3389/fpsyg.2017.00213>.
- Malik, M., Camm, A.J., Bigger, J.T., Breithart, G., Cerutti, S., Cohen, R., 1996. Heart rate variability: standards of measurement, physiological interpretation and clinical use. Task force of the European Society of Cardiology and the north American Society of Pacing and Electrophysiology. Circulation 93 (5), 1043–1065.

**Minor issues:**

**From the abstract – Consideration of the reviewer: The usual nomenclature for a control group in a study focused on electrical stimulation is sham and not placebo, it can be either active sham (electrical current is passed through the test subject) or passive/inactive sham (no current is passed through the test subject)**

**Response:** Thank you very much for your feedback. We have implemented your suggestion and made all the necessary modifications for the sham condition throughout the text.

**From the abstract – Consideration of the reviewer: Better than using “rest” as a way of addressing baseline readings, just use baseline, since the reader immediately knows that you are referring to the period before the stimulation took place, rest could also equally be used for recovery so exactly defining both baseline and recovery is good practice.**

**Response:** Thank you very much; the changes have been made. All instances of the word "rest" in the text have now been replaced with "baseline."

**59-62 Consideration of the reviewer: The sentence does not tell the reader what the “new non-pharmacological adjunct therapy” is, you are probably referring to taVNS, but you should define it, also a source is needed for the whole statement.**

**Response:** Thank you very much. We added a definition of taVNS and included a reference. A minor modification was made to the text. The revised sentence can be found below:

**“**These effects may exacerbate the impairments caused by HIV's direct impact on the central nervous, autonomic, and cardiovascular systems, thereby increasing the risk of developing cardiovascular disease [1,6–9]. Transcutaneous Auricular Vagus Nerve Stimulation (taVNS) is a non-pharmacological adjunct therapy with the potential to help mitigate some of the long-term adverse effects of antiretroviral therapy, particularly regarding cardiac autonomic function) [10].” (Intro, L 56-62).

**REFERENCES**

- Hua, K., Cummings, M., Bernatik, M., Brinkhaus, B., Usichenko, T., & Dietzel, J. (2023). Cardiovascular effects of auricular stimulation -a systematic review and meta-analysis of randomized controlled clinical trials. *Frontiers in Neuroscience*, 17. <https://doi.org/10.3389/fnins.2023.1227858>.

**72. Author: “endothelial cells that form the blood vessels” Consideration of the reviewer: This information is redundant since the endothelium only builds the inner wall of vessels.**

**Response:** Thank you for the observation. The sentence has been revised.

**74. Author: “Furthermore, peripheral nerve fibres can be directly infected by HIV, and through efferent communication, they can reach the central nervous system”. Consideration of reviewer: Did you have retrograde axonal transport in mind? Efferent is the process of going further from the source (in this case the central nervous system) and afferent coming towards the source. Communication in the nervous system is done via electrical (action or graded) potentials, material of any kind cannot be transported in this manner.**

**Response:** Thank you very much for this observation. We have reviewed the text and made it clearer. The modifications can be seen below:

**“**Additionally, HIV can affect the central nervous system through retrograde axonal transport, particularly via the gp120 protein [16]. This mechanism is associated with neurodegeneration and neuronal apoptosis, contributing to the neurological complications observed in patients with HIV [17]”. (Intro, L 76-79).

**REFERENCES**

- Kato, S., Inoue, K., Kobayashi, K., Yasoshima, Y., Miyachi, S., Inoue, S., Hanawa, H., Shimada, T., Takada, M., & Kobayashi, K. (2007). Efficient gene transfer via retrograde transport in rodent and primate brains using a human immunodeficiency virus type 1-based vector pseudotyped with rabies virus glycoprotein. *Human gene therapy*, 18 11, 1141-51. <https://doi.org/10.1089/HUM.2007.082>.
- Kamerman, P., Moss, P., Weber, J., Wallace, V., Rice, A., & Huang, W. (2012). Pathogenesis of HIV‐associated sensory neuropathy: evidence from in vivo and in vitro experimental models. *Journal of the Peripheral Nervous System*, 17. https://doi.org/10.1111/j.1529-8027.2012.00373.x.

**78-82 Author: “Moreover, HIV can induce oxidative stress in the central nervous system, resulting in abnormal signals from sensory receptors”. Consideration of reviewer: Surely oxidative stress in the CNS cannot result in peripheral receptors producing abnormal signals?**

**Response:** Thank you. Following your observation, we reviewed this sentence and decided to remove it. With this change, the revised text now reads as follows:

“In the brain, HIV can cause metabolic disturbances affecting the hypothalamic-pituitary-adrenal axis and the autonomic nervous system, leading to abnormal levels of adrenaline and poor regulation of the cardiovascular system, including changes in heart rate variability (HRV) [18]”. (Intro, L 79-82).

**REFERENCES**

- Chittiprol, S., Shetty, K., Kumar, A., Bhimasenarao, R., Satishchandra, P., Subbakrishna, D., Desai, A., Ravi, V., Satish, K., González, L., & Kumar, M. (2007). HPA axis activity and neuropathogenesis in HIV-1 clade C infection.. *Frontiers in bioscience : a journal and virtual library*, 12, 1271-7 . https://doi.org/10.2741/2145.

**82-84. Author: “Lower resting HRV indices in people with HIV has been associated with vagal activity compared to people without the HIV”. Consideration of reviewer: Probably meant “lower vagal activity”?**

**Response:** Thank you very much. That is exactly what we intended to convey. We have added the word to the text.

**85-87 Author: “Thus, non-pharmacological therapies focusing on autonomic adjustments become necessary and essential for reducing cardiovascular risks as is the case with taVNS”. Consideration of reviewer: Something that hasn’t been proven to be beneficial and you are researching for the first time on a specific population (in your case HIV patients) can’t really be essential, it can be potentially beneficial though.**

**Response:** Perfect. Thank you very much. We have adopted your suggestion and revised the sentence.

“Thus, non-pharmacological therapies focused on autonomic adjustments could potentially be beneficial in reducing cardiovascular risks, as is the case with taVNS.” (Intro, L 85-87).

**91 Author: “According to the neurovisceral integration model, the prefrontal cortex regulates cardiac function through cardiac vagal activity” – Source needed.**

**Response:** Thank you. The source has been added.

**Source added:** According to the neurovisceral integration model, the prefrontal cortex regulates cardiac function through cardiac vagal activity [22].

**REFERENCE**

- Thayer, J., Hansen, A., Saus-Rose, E., & Johnsen, B. (2009). Heart Rate Variability, Prefrontal Neural Function, and Cognitive Performance: The Neurovisceral Integration Perspective on Self-regulation, Adaptation, and Health. *Annals of Behavioral Medicine*, 37, 141-153. <https://doi.org/10.1007/s12160-009-9101-z>.

**97. Author: “However, despite major impairment of cardiovascular activity caused by HIV…” - Source needed.**

**Response:** Thank you. The source has been added.

**Source added:** However, despite major impairment of cardiovascular activity caused by HIV [32], there is a lack of knowledge regarding the effect of taVNS on any clinical outcome in PLHIV.

**REFERENCE**

- Boccara, F., Lang, S., Meuleman, C., Ederhy, S., Mary-Krause, M., Costagliola, D., Capeau, J., & Cohen, A. (2013). HIV and coronary heart disease: time for a better understanding. *Journal of the American College of Cardiology*, 61 5, 511-23 . https://doi.org/10.1016/j.jacc.2012.06.063.

**102-106 Consideration of reviewer: The end of the introduction deserves a more concrete segue into highlighting what the scientific problem is and how exactly does the study that the authors conducted address it.**

**Response:** Thank you very much. We agree with your observation. We revised the text and added another paragraph. The text modifications can be seen below:

“Understanding how taVNS can modulate cardiac autonomic function allows for a potential application of taVNS in reducing cardiovascular risks in people living with HIV, who often exhibit diminished cardiac vagal activity as a consequence of HIV infection and antiretroviral therapy. These autonomic dysfunctions are associated with an increased cardiovascular risk, impacting on the quality of life and longevity of this population. Given that taVNS may positively influence vagal tone, this study aimed to investigate the acute effect of taVNS on cardiac vagal activity in men living with HIV (MLHIV). Therefore, this exploratory investigation assesses whether taVNS can induce an acute vagal response, suggesting its potential as a non-pharmacological intervention to improve autonomic function and reduce cardiovascular risks in PLHIV”. (Intro, L 101-110).

**193 Author: For HRV analysis, low-level artifact correction was applied, the sample length was set at 5 minutes for the HRV process, and a 2% threshold was used for beat corrections. Consideration of reviewer: As someone who has not used Kubios before I do not know what low level artefact correction is and what “2% threshold for beat correction” is, you will need to clarify this for the reader.**

**Response:** Thank you very much. We elaborated further in the text and identified a source for better utilization of Kubios. The modifications can be seen below:

“The RR interval sequence was saved as text files, anonymized by another researcher who modified file names, and later imported into the Kubios HRV Premium software version 3.5.1 (Biosignal Analysis and Medical Imaging Group, Department of Physics, University of Kuopio, Kuopio, Finland) for analysis [48]. The Kubios software was configured to apply low-level artifact correction (0.35 seconds). The algorithm uses a threshold-based detection method, comparing each RR interval to the local average interval calculated through median filtering of the RR interval time series. This approach ensures that the local average is not influenced by outlier RR intervals. RR intervals that deviate from the local average beyond the specified threshold are identified as artifacts and marked for correction by the software [49]. In the present study, the number of corrected heartbeats was less than 2%, as recommended by the Kubios guide, which advises that the percentage of corrected beats should be <5% to avoid significant distortion (suppressed variability) in the analysis results [49]. This process minimizes errors caused by ectopic beats or signal noise, ensuring a cleaner and more accurate dataset for HRV analysis. This approach aligns with the best practices for artifact correction in HRV studies [50,51]”. (Methods, L 233-248).

**REFERENCES**

- Tarvainen, M., Niskanen, J., Lipponen, J., Ranta-Aho, P., & Karjalainen, P. (2014). Kubios HRV - Heart rate variability analysis software. *Computer methods and programs in biomedicine*, 113 1, 210-20. https://doi.org/10.1016/j.cmpb.2013.07.024.
- Tarvainen MP, Lipponen J, Niskanen JP, Ranta-aho PO. Kubios HRV Software USER’S GUIDE. Kubios Oy. 2021. pp. 1–41.
- Laborde, S., Mosley, E., & Thayer, J. (2017). Heart Rate Variability and Cardiac Vagal Tone in Psychophysiological Research – Recommendations for Experiment Planning, Data Analysis, and Data Reporting. Frontiers in Psychology, 8. <https://doi.org/10.3389/fpsyg.2017.00213>.
- Malik, M., Camm, A.J., Bigger, J.T., Breithart, G., Cerutti, S., Cohen, R., (1996). Heart rate variability: standards of measurement, physiological interpretation and clinical use. Task force of the European Society of Cardiology and the north American Society of Pacing and Electrophysiology. Circulation 93 (5), 1043–1065.

**243. Consideration of reviewer: Since you used a generalized linear mixed effect model, I am guessing the distribution of residuals was not normal? It should be mentioned why you chose to use a generalized and not a general mixed effect model. Why did you pick gamma distribution? What was the random effect you mentioned, probably patient, but this should be stated.**

**Response:** Thank you very much for your comment. As we followed the suggestion to reanalyze HRV using the same 5-minute period for all participants, it was necessary to repeat the statistical analysis. In this new approach, we opted to use absolute values without logarithmic transformation. The decision not to apply logarithmic transformation is due to the fact that rMSSD and pNN50 variables exhibited a gamma distribution with right-skewness. Therefore, the statistical modeling was conducted using Generalized Estimating Equations (GEE), which allow for direct adjustment to this distribution without the need for prior data normalization. Additionally, we adopted the GEE approach as it aligns with the longitudinal design of the study and accounts for the dependence of outcome variables as repeated measures. Finally, the GEE analysis was performed considering fixed effects for condition, time, and their interaction. In the first version of the manuscript, the analysis indicated a time effect without an interaction with the stimulation conditions. With the new statistical approach, the results showed no statistically significant differences for time, condition, or their interaction.

“For assumption testing, Q-Q plots of residuals were generated and inspected, and the Shapiro-Wilk normality test was conducted. A t-test was performed to compare baseline variables (SBP and DBP) between the taVNS and sham conditions, with effect sizes calculated using Cohen’s d. A Generalized Estimating Equation (GEE) model for fixed effects (condition, time, condition and time interaction) with a gamma distribution and an identity-link function was selected for the analysis of rMSSD and pNN50, as these variables exhibited a gamma distribution with right-skewness [55,56]. The unstructured covariance matrix was adopted for analysis [57]. It was defined based on the lowest Akaike Information Criterion (AIC) compared to Gaussian distribution. Additionally, residuals were examined to ensure the adequacy of the model assumptions. Effect sizes (Cohen’s d) were calculated for both within- and between-condition comparisons. For between-condition comparisons, Cohen’s d for groups of equal size was used. For within-condition comparisons across time points, Cohen’s d for repeated measures was used and reported as "d repeated measures, pooled." All analyses were performed using Jamovi (Version 2.4), with a significance level set at p < .05 [59–61].” (Methods, L 307-321).

**REFERENCES**

- Park, T., & Shin, D. (1999). On the use of working correlation matrices in the gee approach for longitudinal data. *Communications in Statistics - Simulation and Computation*, 28, 1011-1029. <https://doi.org/10.1080/03610919908813590>.
- De Melo, M., Daldegan-Bueno, D., Oliveira, M., & De Souza, A. (2022). Beyond ANOVA and MANOVA for repeated measures: Advantages of generalized estimated equations and generalized linear mixed models and its use in neuroscience research. *European Journal of Neuroscience*, 56, 6089 - 6098. <https://doi.org/10.1111/ejn.15858>.
- Ballinger, G. (2004). Using Generalized Estimating Equations for Longitudinal Data Analysis. *Organizational Research Methods*, 7, 127 - 150. <https://doi.org/10.1177/1094428104263672>.
- Lakens, D. (2013). Calculating and reporting effect sizes to facilitate cumulative science: a practical primer for t-tests and ANOVAs. *Frontiers in Psychology*, 4. <https://doi.org/10.3389/fpsyg.2013.00863>.
- The jamovi project. Jamovi; 2023. Available: <https://www.jamovi.org>.
- R Core Team. R: A Language and environment for statistical computing. 2022. Available: <https://cran.r-project.org>.
- Kerby, D. S. (2014). The simple difference formula: An approach to teaching nonparametric correlation. Comprehensive Psychology, 3, 11-IT. <http://doi:10.2466/11.it.3.1>

**294 Author: “From the second recovery measurement (30th min) to rest…” Consideration of reviewer: Implies you only used a single minute of RR intervals to calculate HRV.**

**Response:** Thank you very much for your comment. To ensure standardization across all participants, we selected the same 5-minute periods within each of the six 10-minute windows. Specifically, we adopted the first 5 minutes of each 10-minute window for HRV calculations. Based on your comment, we also realized that our figure could be more informative, so we have made adjustments accordingly. The updated text can be seen below:

**“**A total of six 10-minute segments were extracted from the timeline for analysis: Moment 1 (baseline), Moments 2 and 3 during stimulation (0-10 min and 20-30 min), and Moments 4, 5, and 6 during recovery (30-40 min, 50-60 min, and 80-90 min). Within each 10-minute window corresponding to these moments, HRV was analyzed in the first 5 minutes across both conditions and for all participants to ensure better data comparability.” (Methods, L 249-253).

**344 Author: “The use of the earlobe as an active simulated taVNS stimulation is common, and the choice of this structure is based on imaging studies [46–48] which in turn used the tragus for taVNS, and a dissection study [49].” In this section you address the control as “active simulated taVNS”, further in the manuscript you address it as “simulated stimulation” (line 355) while in the beginning it was “placebo”. The correct terminology is passive sham or active sham.**

**Response:** Thank you very much. We have modified the terms throughout the entire text.

**346 Author: “Peuker e Filler”. Consideration of reviewer: Did you mean “Peuker and Filler”?**

**Response:** That's right. We have corrected it. Thank you very much.

**358 Author: “We can suppose that the absence of differences was due to the choice of other parameters involved in the stimulation protocol, such as intensity. Consideration of reviewer: It is not really scientific to assume what the cause of some effect was in a study that isn’t ours. We can state the facts and hypothesize, but never assume.**

**Response:** Thank you very much. We have modified the beginning of the paragraph; the new sentence can be seen below.

**“**The absence of differences may be influenced by various parameters of the stimulation protocol, including intensity.” (Discussion, L 426-427).

**365-378 Consideration of reviewer: This section focuses on the comparison of stimulation intensities across various studies. Stimulation intensities are not directly comparable among studies due to the significant variations in measurement methods; most researchers typically depend on the stimulator's display readout, which isn’t a standardized or accurate measurement. Consequently, the reported average stimulation currents vary among studies. Furthermore, even if all studies employed precise current sensing probes, the differing current waveforms (such as biphasic versus monophasic) would elicit a different sensation of stimulation for participants, necessitating different currents for each waveform to achieve a somewhat similar experience.**

**Response:** Thank you very much for this observation. We have revised the paragraph, modifying some sentences. Additionally, we have incorporated a reflection based on your consideration, along with some references to support the argument.

“For intensity adjustment, we followed the recommendations proposed by Badran et al., [43]. Volunteers who reported discomfort had their intensity immediately reduced, but not necessarily below the perceptual threshold, as vagus nerve activation is suggested to occur between the perceptual and pain thresholds [71]. The mean intensity used in this study was 8 mA, which falls within the range of previous taVNS studies [72,73]. For example, Geng et al., [74] reported a mean intensity of 16 mA and found significant differences in rMSSD between conditions (taVNS vs. sham) and in the condition × time interaction; however, taVNS was applied to the tragus. Some studies suggest that higher intensities may enhance neuromodulatory effects [75]. However, it has been shown that higher intensity can lead to greater discomfort, which is not associated with higher values of rMSSD [62], whereas others report significant changes in HRV even at lower intensities (e.g., 1.2 mA) [24]. It is important to note that direct comparisons of stimulation intensities across studies should be interpreted with caution due to methodological variations. Studies report intensities based on device readings, which may not be standardized or directly comparable. Despite some attempts to investigate the use of different stimulation parameters [62], different current waveforms (such as biphasic vs. monophasic) may elicit distinct stimulation sensations for participants, particularly when the stimulation site differs (tragus vs. cymba conchae) [76,77], requiring different current levels for each waveform to achieve a comparable sensory experience. Given these discrepancies, we recommend that future studies conduct more in-depth investigations to systematically explore the interaction between stimulation intensity, waveform characteristics, electrode placement in different ear regions, and, consequently, skin thickness assessment”. (Discussion, L 4270-449).

**REFERENCES**

- Badran, B., Yu, A., Adair, D., Mappin, G., Devries, W., Jenkins, D., George, M., & Bikson, M. (2019). Laboratory Administration of Transcutaneous Auricular Vagus Nerve Stimulation (taVNS): Technique, Targeting, and Considerations. *Journal of visualized experiments: JoVE*, 143. <https://doi.org/10.3791/58984>.
- Yuan, H., & Silberstein, S. (2016). Vagus Nerve and Vagus Nerve Stimulation, a Comprehensive Review: Part II. Headache: The Journal of Head and Face Pain, 56. https://doi.org/10.1111/head.12650.
- Weise, D., Adamidis, M., Rumpf, J., Fricke, C., & Classen, J. (2015). P184. Heart rate variability and its modulation by auricular branch of vagus nerve stimulation in Parkinson’s disease. Clinical Neurophysiology, 126. <https://doi.org/10.1016/j.clinph.2015.04.280>.
- Keute, M., Ruhnau, P., Heinze, H., & Zaehle, T. (2018). Behavioral and electrophysiological evidence for GABAergic modulation through transcutaneous vagus nerve stimulation. *Clinical Neurophysiology*, 129, 1789-1795. <https://doi.org/10.1016/j.clinph.2018.05.026>.
- Geng, D., Liu, X., Wang, Y., & Wang, J. (2022). The effect of transcutaneous auricular vagus nerve stimulation on HRV in healthy young people. *PLoS ONE*, 17. <https://doi>.org/10.1371/ornal.pone.0263833.
- Mertens, A., Carrette, S., Klooster, D., Lescrauwaet, E., Delbeke, J., Wadman, W., Carrette, E., Raedt, R., Boon, P., & Vonck, K. (2021). Investigating the Effect of Transcutaneous Auricular Vagus Nerve Stimulation on Cortical Excitability in Healthy Males. *Neuromodulation: journal of the International Neuromodulation Society*. https://doi.org/10.1111/ner.13488.
- Borges, U., Laborde, S., & Raab, M. (2019). Influence of transcutaneous vagus nerve stimulation on cardiac vagal activity: Not different from sham stimulation and no effect of stimulation intensity. *PloS ONE*, 14. <https://doi.org/10.1371/journal.pone.0223848>.
- Forte, G., Favieri, F., Leemhuis, E., De Martino, M., Giannini, A., De Gennaro, L., Casagrande, M., & Pazzaglia, M. (2022). Ear your heart: transcutaneous auricular vagus nerve stimulation on heart rate variability in healthy young participants. *PeerJ*, 10. <https://doi.org/10.7717/peerj.14447>.
- Kaniušas, E., Kampusch, S., Tittgemeyer, M., Panetsos, F., Ginés, R., Papa, M., Kiss, A., Podesser, B., Cassarà, A., Tanghe, E., Samoudi, A., Tarnaud, T., Joseph, W., Marozas, V., Lukoševičius, A., Ištuk, N., Lechner, S., Klonowski, W., Varoneckas, G., Szeles, J., & Šarolić, A. (2019). Current Directions in the Auricular Vagus Nerve Stimulation II – An Engineering Perspective. *Frontiers in Neuroscience*, 13. <https://doi.org/10.3389/fnins.2019.00772>.
- Burger, A., D’Agostini, M., Verkuil, B., & Van Diest, I. (2020). Moving beyond belief: A narrative review of potential biomarkers for transcutaneous vagus nerve stimulation.. *Psychophysiology*, e13571 . <https://doi>.org/10.1111/psyp.13571.

**387 Authors: “Studies have shown relaxing effects of virtual environments, such as watching nature films, on the autonomic nervous system, measured by heart rate variability [60].” Consideration of reviewer: Just a thought, if displaying visual content from a natural environment produces comparable results to taVNS, maybe an intervention using virtual reality and natural landscapes would make sense for a future study.**

**Response:** Thank you for your observation. After reanalyzing the statistical data while taking your considerations into account, the differences previously identified over time were no longer observed. Therefore, we decided to remove this paragraph from the discussion. However, we would like to highlight that we carefully selected videos of a similar style and content that did not evoke emotions capable of influencing HRV. Consequently, we did not anticipate any changes. Thus, after considering your observation, we recognize this as a limitation, as we did not use empirically tested/validated neutral videos. For this reason, we have decided to include this limitation, which can be found below.

**“**Participants in the study spent the entire session seated on a couch, watching POV-style videos of streets in different countries. Care was taken to select videos of the same style and content to avoid evoking emotions such as anger, sadness, or euphoria. However, this aspect was not controlled in the study, as the videos had not been empirically tested or validated as neutral.” (Limitations, L 511-515).

**388 Authors: It is possible that HRV may not be an ideal biological marker for measuring the modulation of cardiac vagal activity in taVNS studies[61]. Consideration of reviewer: Very valid and important point.**

**Response:** We are pleased with your comment. Thank you very much.

**399 Authors: “Although we conducted a randomized, crossover clinical trial to reduce sample heterogeneity, it is still possible that in HIV there are relevant factors that need to be investigated, such as the amount of virus in the nervous system…” Consideration of reviewer: this is a very important consideration I am glad that you mentioned it.**

**Response:** Thank you very much. We are even happier.

**406 Authors: “The absence of significant differences of taVNS on HRV can be explained by the first activation pathway, where taVNS operates in an indirect and complex system of brain regions and nuclei[65]”. Consideration of reviewer: Did you mean the absence of effect of taVNS on HRV? What is the first activation pathway, I tried finding it in the source you provided but was unsuccessful.**

**Response:** Thank you for your observation. We have made modifications to the paragraph to enhance its clarity for the reader. Additionally, we have included some references that provide a better explanation of the possible pathways of the taVNS activation mechanism in the cardiovascular system.

“The absence of taVNS effects on cardiac vagal activity may be attributed to the complex mechanisms underlying taVNS activation in the cardiovascular system. taVNS appears to excite afferent fibers that do not directly innervate the heart but may indirectly modulate it via brainstem nuclei [76]. This process enhances the input to the nucleus tractus solitarius (NTS) and influences the activity of NTS neurons that project to vagal cardioinhibitory efferent neurons located in the dorsal vagal nucleus (DVN) and the nucleus ambiguus (NA). These vagal efferent neurons propagate vagal tone to the sinoatrial (SA) node [84]. Additionally, taVNS may also excite NTS neurons that send excitatory projections to the caudal ventrolateral medulla (CVLM), which inhibits the rostral ventrolateral medulla (RVLM) – the primary source of excitatory drive to sympathetic pre-ganglionic neurons in the intermediolateral cell column (IML) of the spinal cord [21]”. (Discussion, L 474-485).

**REFERENCES**

- Kaniušas, E., Kampusch, S., Tittgemeyer, M., Panetsos, F., Ginés, R., Papa, M., Kiss, A., Podesser, B., Cassarà, A., Tanghe, E., Samoudi, A., Tarnaud, T., Joseph, W., Marozas, V., Lukoševičius, A., Ištuk, N., Lechner, S., Klonowski, W., Varoneckas, G., Szeles, J., & Šarolić, A. (2019). Current Directions in the Auricular Vagus Nerve Stimulation II – An Engineering Perspective. *Frontiers in Neuroscience*, 13. <https://doi.org/10.3389/fnins.2019.00772>.
- Murray, A., Atkinson, L., Mahadi, M., Deuchars, S., & Deuchars, J. (2016). The strange case of the ear and the heart: The auricular vagus nerve and its influence on cardiac control. Autonomic Neuroscience, 199, 48-53. <https://doi.org/10.1016/j.autneu.2016.06.004>.
- Butt, M., Albusoda, A., Farmer, A., & Aziz, Q. (2019). The anatomical basis for transcutaneous auricular vagus nerve stimulation. Journal of Anatomy, 236. <https://doi.org/10.1111/joa.13122>.

**451. Authors: “Unlike some previous studies that reported positive effects of taVNS on rMSSD and pNN50 in healthy individuals” …” Consideration of reviewer: This must be understood in context; although numerous studies indicate a change in HRV during taVNS, this is only accurate when comparing baseline HRV to HRV during taVNS. When comparing HRV during taVNS against sham in both healthy individuals and patients with different conditions, the effect is mostly non-existent, except for the LF/HF HRV index, which does show change. (Hua et al., 2023; Wolf et al. 2021).**

**Response:** Thank you very much for this suggestion. We have incorporated the suggested information into the paragraph in the Discussion section. The changes can be seen below:

**“**Although several studies report changes in HRV during taVNS, these alterations are observed only when comparing baseline HRV to HRV during stimulation. However, when comparing HRV between taVNS and sham stimulation in healthy individuals and patients with different clinical conditions, the effect is mostly non-existent, except for the LF/HF index [10,77]. Nevertheless, it is important to highlight that the LF/HF ratio is not an ideal marker of cardiac vagal activity, which limits its interpretability [50,51]. Still, certain patient groups, particularly those with differences in baseline cardiac vagal activity, may exhibit distinct responses to taVNS [77,78]”. (Discussion, L 456-463).

**REFERENCES**

- Hua, K., Cummings, M., Bernatik, M., Brinkhaus, B., Usichenko, T., & Dietzel, J. (2023). Cardiovascular effects of auricular stimulation -a systematic review and meta-analysis of randomized controlled clinical trials. *Frontiers in Neuroscience*, 17. <https://doi.org/10.3389/fnins.2023.1227858>.
- Wolf, V., Kühnel, A., Teckentrup, V., Koenig, J., & Kroemer, N. (2021). Does transcutaneous auricular vagus nerve stimulation affect vagally mediated heart rate variability? A living and interactive Bayesian meta-analysis. Psychophysiology, e13933. https://doi.org/10.1111/psyp.13933.
- Laborde, S., Mosley, E., & Thayer, J. (2017). Heart Rate Variability and Cardiac Vagal Tone in Psychophysiological Research – Recommendations for Experiment Planning, Data Analysis, and Data Reporting. *Frontiers in Psychology*, 8. <https://doi.org/10.3389/fpsyg.2017.00213>.
- Malik, M., Camm, A.J., Bigger, J.T., Breithart, G., Cerutti, S., Cohen, R., (1996). Heart rate variability: standards of measurement, physiological interpretation and clinical use. Task force of the European Society of Cardiology and the north American Society of Pacing and Electrophysiology. Circulation 93 (5), 1043–1065.
- Soltani D, Azizi B, Sima S, Tavakoli K, Hosseini Mohammadi NS, Vahabie A-H, et al. A systematic review of the effects of transcutaneous auricular vagus nerve stimulation on baroreflex sensitivity and heart rate variability in healthy subjects. (2023). Clinical Autonomic Research.;33: 165–189. <http://doi:10.1007/s10286-023-00938-w>.

**Table 1. Consideration of reviewer: Nicely presented table.**

**Response:** Thank you very much. We're glad you liked it.

**Figure 3. Consideration of reviewer: This figure needs additional information on the x axis. As I understand it, the time stamp indicators on the X axis represent periods, yet in their current form, they just appear as moments in time. Additionally, the repetition of these markers adds to the confusion. The x axis also needs to be relabeled to ECG or RR since that is the signal that was being measured. HRV is a calculated index not a measurement.**

**Response:** Thank you very much. We've taken your suggestion and made a new figure 3. We've increased the detail of the time division, the 10-minute windows used for RR interval recording clippings and the first 5 minutes of each window used for heart rate variability analysis.

Hua, K., Cummings, M., Bernatik, M., Brinkhaus, B., Usichenko, T., & Dietzel, J. (2023). Cardiovascular effects of auricular stimulation -a systematic review and meta-analysis of randomized controlled clinical trials. Frontiers in Neuroscience, 17(September), 1–18. https://doi.org/10.3389/fnins.2023.1227858

Wolf, V., Kühnel, A., Teckentrup, V., Koenig, J., & Kroemer, N. B. (2021). Does transcutaneous auricular vagus nerve stimulation affect vagally mediated heart rate variability? A living and interactive Bayesian meta-analysis. Psychophysiology, 58(11). https://doi.org/10.1111/psyp.13933

**REVIEWER 3**

**Reviewer #3: General comments:**

**This study evaluated a randomized trial of transcutaneous auricular vagus nerve stimulation (taVNS) on cardiac vagal activity in people living with HIV.**

**I found a number of methodological points for which I have concerns and have listed those in the specific comments below. I recommend giving some thought to how the methods are explained and how the results are reported, because I found both confusing and somewhat lacking.**

**Overall, I feel like 21 participants, even in a crossover trial, is probably not enough to achieve robust results. Thus, I think you should lean more heavily toward labeling this a proof-of-concept study rather than expecting this to demonstrate results that are more definitive than that. I feel as though the authors do this a little bit in the discussion, but I would have preferred a stronger lean in that direction.**

**Response:** Thank you very much for your comments. We have taken on board your suggestion and decided to label the study as a proof of concept. We have explicitly incorporated the term "proof-of-concept" in the relevant sections of the article's structure. Additionally, the title has been updated accordingly and now reads: *“Acute effect of transcutaneous auricular vagus nerve stimulation on cardiac vagal activity in men living with HIV: a proof-of-concept clinical trial.”*

**Specific comments:**

1. **(lines 111-117) I am confused about how the power analyses are reported here. For almost any trial, power analyses are a required and necessary step. Extenuating circumstances happen and sometimes power and sometimes the desired sample sizes are not met. Sure, that's suboptimal, but it's ok. I don't understand why an after-the-fact power calculation is reported instead. Post-hoc or retrospective power has some controversy and there are divided opinions on the worth of post-hoc power. I think post-hoc power can be used to emphasize end of study conclusions, but I don't find this use of post-hoc power appropriate. Please include the pre-study power analyses and use the section to discuss why the sample size was not met. I think you partially do this on lines 130-135.**

**Response:** We greatly appreciate your comment. We agree that the current sample size may not be sufficient to detect significant effects on the primary clinical outcomes. Regarding the use of the term "post-hoc," we would like to clarify that there was an error on our part in the description. We are grateful for the opportunity to explain in detail how we conducted the sample size calculation using G*Power. The sample size calculation was conducted based on a priori analysis for repeated measures analysis of variance. We used an effect size of f = 0.24, based on the study by Vosseler et al., (2020), which employed a similar stimulation protocol to ours. The following input parameters were adopted: α error probability of 0.05, power of 0.80, two groups, six measurements, and a correlation among repeated measures of 0.5. The calculated sample size was 20 participants. However, we agree that, due to the specific variables and the studied population, it would be more prudent to increase the number of participants. We managed to recruit 35 participants, but unfortunately, only 21 met all inclusion criteria. Therefore, the suggestion to refer to the study as a proof of concept makes complete sense. We thank you again for your observation, and below are the modifications made to the manuscript based on your comment.

**“**A priori sample size calculation was conducted using effect size f = 0.24 for repeated measures analysis of variance with between factors based on previous study [37]. The following input parameters were adopted: α error probability of 0.05, power of 0.80, two number of groups, six number of measurements, and correlation among repeated measures of 0.5. The total sample size calculated was defined as 20 participants. The sample size calculation was performed using the software G*Power [38]”. (Methods, L 121-127).

“Additionally, despite extensive efforts to recruit participants in hospitals and collaborate with infectious disease specialists, we encountered significant challenges in achieving a larger sample size. A total of 35 individuals were assessed for eligibility; however, 14 did not meet the inclusion criteria, resulting in a final sample of 21 participants. While we acknowledge that a larger sample size could provide more robust estimates, we believe that the data obtained contribute to a better understanding of the potential acute effects of transcutaneous auricular vagus nerve stimulation (taVNS) in PLHIV, serving as a foundation for future investigations.” (Methods, L 145-152).

**REFERENCE**

- Vosseler, A., Zhao, D., Fritsche, L., Lehmann, R., Kantartzis, K., Small, D., Peter, A., Häring, H., Birkenfeld, A., Fritsche, A., Wagner, R., Preissl, H., Kullmann, S., & Heni, M. (2020). No modulation of postprandial metabolism by transcutaneous auricular vagus nerve stimulation: a cross-over study in 15 healthy men. *Scientific Reports*, 10. <https://doi.org/10.1038/s41598-020-77430-2>.

2. **(lines 239-241) I strongly encourage the authors to use standardized mean differences instead of p-values to compare baseline and 12-month characteristics. Significance testing in these situations is generally frowned upon because a non-significant p-value does not indicate that groups are the same. For info on the topic in relation to baseline imbalance in randomized trials see Altman,** [**https://doi.org/10.2307/2987510**](https://doi.org/10.2307/2987510) **and Senn,** [**https://doi.org/10.1002/sim.4780131703**](https://doi.org/10.1002/sim.4780131703)**. My recommendation is to use standardized difference to assess differences (see Austin, https://doi.org/10.1080/03610910902859574).**

**Response:** Thank you very much. We could not understand the reference to "12 months," as our experimental design did not incorporate this timeframe. However, we believe the suggestion pertains to the time points (stimulation and recovery) in relation to the baseline. Accordingly, we have incorporated your suggestion and created a new table (*Table 3*), where we present the standardized mean differences for all HRV analysis time points. Additionally, we calculated the effect size for repeated measures of each variable (*rMSSD* and *pNN50*), comparing them to the baseline in both the *taVNS* and *sham* groups. We also computed the effect size between stimulation conditions at each time point. Finally, we calculated the pooled effect size across conditions. All these details can be found in the updated table.

3. **(lines 241-245) Without a methodological citation, I am not sure what statistical method is being used. I presume you have run a generalized linear mixed model (GLMM) with because you have assumed a Gamma distribution. But, you said that you've used an identity link function despite the canonical link function for a Gamma being a log link. Please describe in further detail the methods used here with methodological citations and indicate why you have chosen not to use the canonical link function.**

**Response:** Thank you very much for your comment. As we followed the suggestion to reanalyze HRV using the same 5-minute period for all participants, it was necessary to repeat the statistical analysis. In this new approach, we opted to use absolute values without logarithmic transformation. The decision not to apply logarithmic transformation is due to the fact that rMSSD and pNN50 variables exhibited a gamma distribution with right-skewness. Therefore, the statistical modeling was conducted using Generalized Estimating Equations (GEE), which allow for direct adjustment to this distribution without the need for prior data normalization. Additionally, we adopted the GEE approach as it aligns with the longitudinal design of the study and accounts for the dependence of outcome variables as repeated measures. Finally, the GEE analysis was performed considering fixed effects for condition, time, and their interaction. In the first version of the manuscript, the analysis indicated a time effect without an interaction with the stimulation conditions. With the new statistical approach, the results showed no statistically significant differences for time, condition, or their interaction.

“For assumption testing, Q-Q plots of residuals were generated and inspected, and the Shapiro-Wilk normality test was conducted. A t-test was performed to compare baseline variables (SBP and DBP) between the taVNS and sham conditions, with effect sizes calculated using Cohen’s d. A Generalized Estimating Equation (GEE) model for fixed effects (condition, time, condition and time interaction) with a gamma distribution and an identity-link function was selected for the analysis of rMSSD and pNN50, as these variables exhibited a gamma distribution with right-skewness [55,56]. The unstructured covariance matrix was adopted for analysis [57]. It was defined based on the lowest Akaike Information Criterion (AIC) compared to Gaussian distribution. Additionally, residuals were examined to ensure the adequacy of the model assumptions. Effect sizes (Cohen’s d) were calculated for both within- and between-condition comparisons. For between-condition comparisons, Cohen’s d for groups of equal size was used. For within-condition comparisons across time points, Cohen’s d for repeated measures was used and reported as "d repeated measures, pooled." All analyses were performed using Jamovi (Version 2.4), with a significance level set at p < .05 [59–61].” (Methods, L 307-321).

**REFERENCES**

- Park, T., & Shin, D. (1999). On the use of working correlation matrices in the gee approach for longitudinal data. *Communications in Statistics - Simulation and Computation*, 28, 1011-1029. <https://doi.org/10.1080/03610919908813590>.
- De Melo, M., Daldegan-Bueno, D., Oliveira, M., & De Souza, A. (2022). Beyond ANOVA and MANOVA for repeated measures: Advantages of generalized estimated equations and generalized linear mixed models and its use in neuroscience research. *European Journal of Neuroscience*, 56, 6089 - 6098. <https://doi.org/10.1111/ejn.15858>.
- Ballinger, G. (2004). Using Generalized Estimating Equations for Longitudinal Data Analysis. *Organizational Research Methods*, 7, 127 - 150. <https://doi.org/10.1177/1094428104263672>.
- Lakens, D. (2013). Calculating and reporting effect sizes to facilitate cumulative science: a practical primer for t-tests and ANOVAs. *Frontiers in Psychology*, 4. <https://doi.org/10.3389/fpsyg.2013.00863>.
- The jamovi project. Jamovi; 2023. Available: <https://www.jamovi.org>.
- R Core Team. R: A Language and environment for statistical computing. 2022. Available: <https://cran.r-project.org>.
- Kerby, D. S. (2014). The simple difference formula: An approach to teaching nonparametric correlation. Comprehensive Psychology, 3, 11-IT. <http://doi:10.2466/11.it.3.1>

4. **(line 243) I don't know what a "non-random covariance matrix" is. Again, please provide a methodological citation for this so I can better understand the method implemented.**

**Response:** Thank you very much. As mentioned in the previous comment, our analysis was revised using *Generalized Estimating Equation* (*GEE*) models. In this new analysis, we employed an unstructured covariance matrix, following the recommendation of the reference cited below. On page 133, a more detailed explanation of the covariance matrix can be found.

**REFERENCES**

- Ballinger, G. (2004). Using Generalized Estimating Equations for Longitudinal Data Analysis. *Organizational Research Methods*, 7, 127 - 150. <https://doi.org/10.1177/1094428104263672>.

5. **(line 291) I don't recall information in the methods section regarding a mediation analysis. How did you evaluation mediation?**

**Response:** Thank you very much. We acknowledge that we may have used this term inappropriately in the results section. We would like to inform you that the term has been removed from the text.

6. **(line 293 and onward) What is the interpretation of these effects? Are they mean differences between the treatment conditions? Are they differences in the slopes? If this is a Gamma regression, then maybe they are arithmetic mean ratios? Apologies, but without units on these effect sizes nor description, I am not able to interpret these numbers.**

**Response:** Thank you very much. The data were reanalyzed using the original, non-log-transformed measures and applying the Generalized Estimating Equations (GEE), which is the most appropriate method for these data. Descriptive data and mean differences are presented in Table 3. The text interpretation focuses solely on the main effects of time, condition, and the time-condition interaction as analyzed by GEE. The results indicate no statistically significant main effects of time or condition, despite a marginal mean difference of 2.08 ms for rMSSD and 2.23% for pNN50 between conditions. Additionally, the slopes were not significantly different, as demonstrated by the time-condition interaction. We also added a paragraph discussing the results of the effect size calculations. Finally, Table 3 presents the descriptive means with their respective 95% confidence intervals (CIs), the standardized mean differences between conditions at each time point, as well as all effect size calculations. After reanalyzing the data, the results have been described as shown in the sentence below:

“The GEE revealed no significant effects of condition (rMSSD: χ²(1) = .049, *p* = .484; pNN50: χ²(1) = 1.160, *p* = .281), time (rMSSD: χ²(5) = 5.81, p = 0.325; pNN50: χ²(5) = 6.250, *p* = .283), or condition-by-time interaction (rMSSD: χ²(5) = .260, *p* = .998; pNN50: χ²(5) = .130, *p* = 1.000) for rMSSD or pNN50, indicating taVNS did not significantly alter these measures compared to sham over time. Effect sizes (Cohen’s *d*) for within-condition changes relative to baseline ranged from moderate to large for rMSSD and pNN50 in taVNS, and from small to moderate (rMSSD) or moderate to large (pNN50) in sham, with similar trends over time. Between-condition effect sizes were consistently small at each time point, with overall effect sizes of *d* = 0.52 (rMSSD) and *d* = 0.78 (pNN50), indicating moderate effects. Marginal effects of taVNS versus sham were nonsignificant for rMSSD (2.08 ms, 95% CI: -3.77 to 7.99 ms, Z = 0.69, *p* = .485) and pNN50 (2.23%, 95% CI: -1.84 to 6.37%, Z = 1.08, *p* = .282). Descriptive results are detailed in Table 3.” (Results, L 355-367).
